# Deacetylation biocatalysis and elicitation by immobilized *Penicillium canescens* in *Astragalus membranaceus* hairy root cultures: towards the enhanced and sustainable production of astragaloside IV

**DOI:** 10.1111/pbi.12612

**Published:** 2016-09-16

**Authors:** Qing‐Yan Gai, Jiao Jiao, Meng Luo, Wei Wang, Li‐Ping Yao, Yu‐Jie Fu

**Affiliations:** ^1^ Key Laboratory of Forest Plant Ecology Ministry of Education Northeast Forestry University Harbin China; ^2^ Engineering Research Center of Forest Bio‐Preparation Ministry of Education Northeast Forestry University Harbin China; ^3^ Collaborative Innovation Center for Development and Utilization of Forest Resources Harbin Heilongjiang China

**Keywords:** *Penicillium canescens*, biocatalysis, elicitation, astragaloside IV, hairy root cultures

## Abstract

A novel biotechnology approach by combining deacetylation biocatalysis with elicitation of immobilized *Penicillium canescens* (IPC) in *Astragalus membranaceus* hairy root cultures (AMHRCs) was proposed for the elevated production of astragaloside IV (AG IV). The highest AG IV accumulation was achieved in 36‐day‐old AMHRCs co‐cultured with IPC for 60 h, which resulted in the enhanced production of AG IV by 14.59‐fold in comparison with that in control (0.193 ± 0.007 mg/g DW). Meanwhile, AG IV precursors were almost transformed to AG IV by IPC deacetylation. Moreover, expression of genes involved in AG IV biosynthetic pathway was significantly up‐regulated in response to IPC elicitation. Also, FTIR and SEM showed that cell wall lignification was enhanced following IPC treatment and root surface was likely to be IPC deacetylation site. Overall, dual roles of IPC (biocatalyst and elicitor) offered an effective and sustainable way for the mass production of AG IV in AMHRCs.

## Introduction

**Figure 1:**
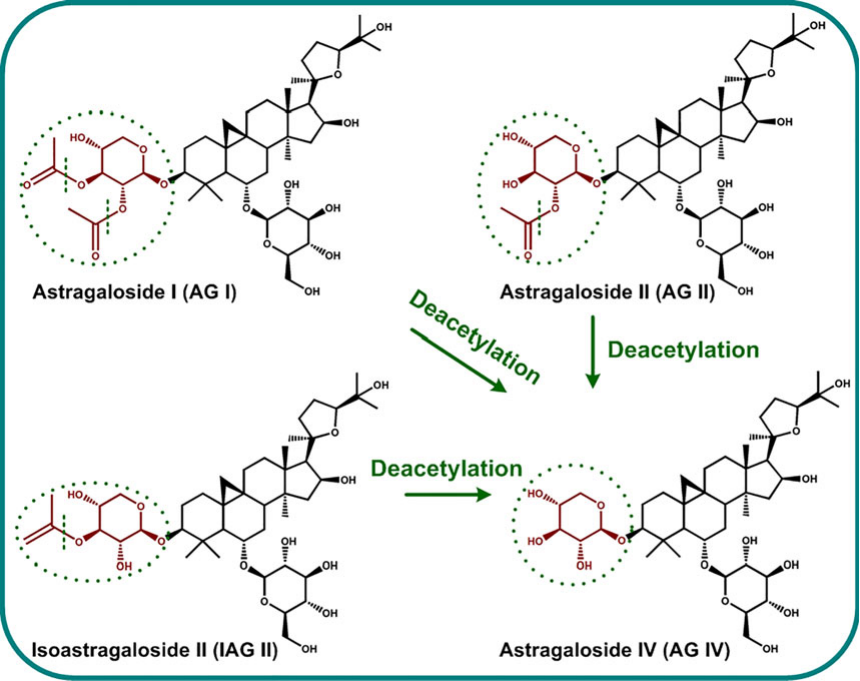
Biotransformation of AG IV precursors  (AG I, AG II and IAG II) to AG IV by deacetylation biocatalysis.

Astragalosides (AGs) are a class of cycloartane‐type triterpene saponins mainly existing in *Astragalus membranaceus* roots, *for example* astragaloside I (AG I), astragaloside II (AG II), isoastragaloside II (IAG II), astragaloside III (AG III) and astragaloside IV (AG IV) (Ionkova *et al*., 2014). Among them, AG IV has received tremendous interest due to its multiple health benefits as diverse as cardioprotective, antitumour, antiviral, immunoregulatory, anti‐inflammatory, hepatoprotective, antidiabetic and neuroprotective activities (Ionkova *et al*., 2014; Li *et al*., 2014; Ren *et al*., 2013). Unfortunately, the chemical synthesis of AG IV is quite difficult and commercially infeasible due to the complicated stereochemical ring with multiple chiral centres (Figure 1). Currently, the low production and fitful supply of AG IV from natural or cultivated *A. membranaceus* sources present a significant limit for the large‐scale industrial usage of AG IV in drug development (Ionkova *et al*., 2014; Ma *et al*., 2002).

Recent efforts have been made to develop plant cell/organ cultures as viable alternative production platforms for valuable phytochemicals that can be scaled up in bioreactors under controlled conditions (Murthy *et al*., 2014). In this context, we have developed *A. membranaceus* hairy root cultures (AMHRCs) as a promising biotechnology system that can supersede field‐grown plants for the sustainable production of AGs (Jiao *et al*., 2015). However, the content of AG IV in AMHRCs is still relatively low, *ca*. 0.02% dry weight (DW). Factually, some major AG components with low bioactivity, *for example* AG I, AG II and IAG II, are structurally similar to AG IV, but possess extra acetyl residues attached at C‐3 position (Figure 1). Recently, microbial biotransformation has been recognized to be superior to conventional chemical procedures for the hydrolysis of unnecessary acetyl residues in these precursors to produce AG IV, owing to its highly catalytic efficiency, inherent selectivity, mild conditions, environmentally friendliness, low cost and simple downstream processing (Yao *et al*., 2014; Ye *et al*., 2011). However, the substrates used in these microbial biotransformation processes are crude AG extracts or *A. membranaceus* dried roots. Whether microbial biotransformation can enhance AG IV production in live plant *in vitro* cultures, *that is* AMHRCs, is still unknown.

**Figure 2:**
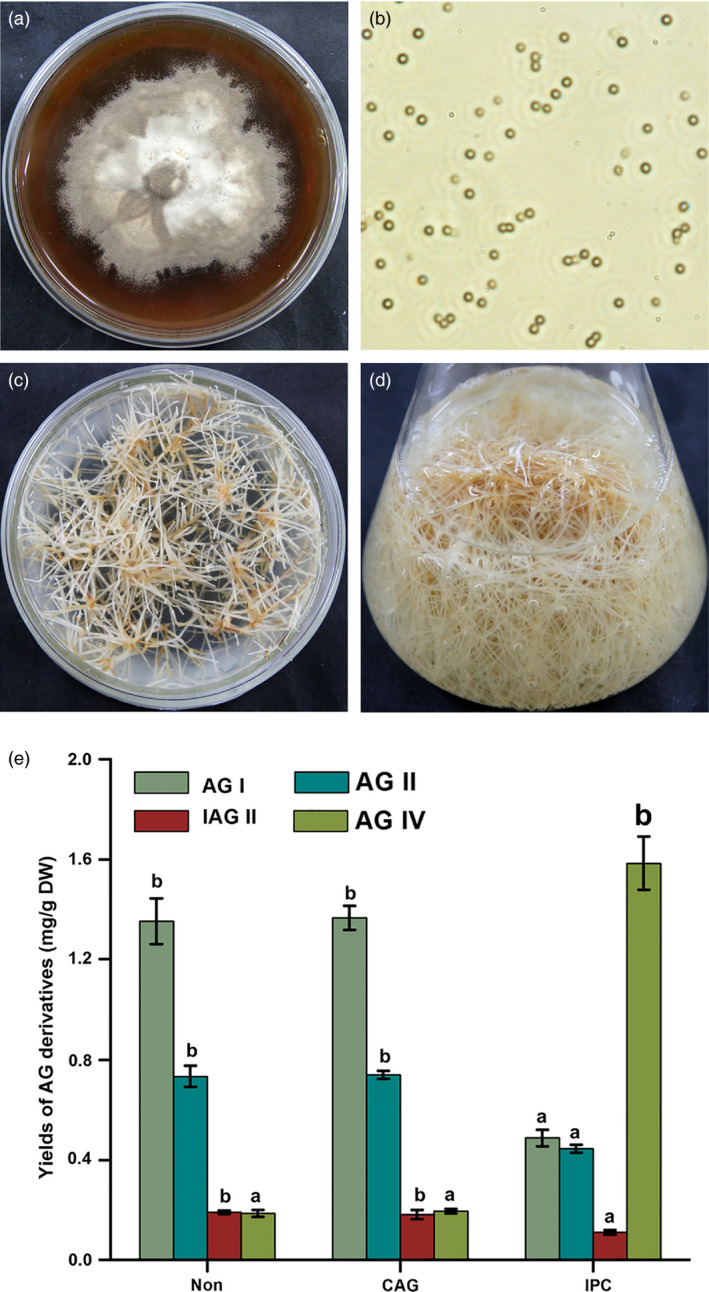
(a) *Penicillium canescens* colony on PDA medium; (b) photomicrograph of *P. canescens* spores; (c) a high‐productive AMHRL VI on MS solid medium; (d) AMHRCs in MS liquid medium; (e) effects of nontreatment, CAG and IPC treatments on yields of different AG derivatives in 36‐day‐old AMHRCs (*ca*. 5 × 10^2^ spores/mL, co‐cultivation temperature 28 °C, initial pH value of media 5.8 and time 48 h). Non, nontreated AMHRCs; CAG, CAG‐treated AMHRCs; IPC, IPC‐treated AMHRCs. Mean ± SD values not sharing the same lowercase letters are significantly different (*P* < 0.05).

In our previous report, a special endophytic fungus *Penicillium canescens* (Figure 2a) isolated from pigeon pea is found to possess strong deacetylation ability that can effectively transform other AG derivatives to AG IV (Yao *et al*., 2014). Therefore, an interesting possibility is to exploit the deacetylation biocatalysis of *P. canescens* for enhancing AG IV production in AMHRCs. Moreover, triterpene saponins are a class of secondary metabolites that can contribute to plant defence against a broad spectrum of insects, pathogens and other herbivores (Augustin *et al*., 2011; Khakimov *et al*., 2015; Moses *et al*., 2015). Based on this principle, AG IV biosynthesis is sensitive to fungus attacks and is strongly inducible. In this regard, the elicitation effect of *P. canescens* can also trigger plant defence metabolisms for further promoting AG IV accumulation in AMHRCs.

Based on the foregoing, we established a co‐cultivation system of plant–fungus for the elevated production of AG IV *via* coupled culture of AMHRCs and immobilized *P. canescens* (IPC). The feasibility of IPC for promoting AG IV production in live AMHRCs was initially investigated. Subsequently, the biotransformation time course of AG IV and its precursors (AG I, AG II and IAG II) in AMHRCs was monitored. To understand the elicitation mechanism of IPC, transcriptional profiles of associated genes involved in AG IV biosynthetic pathway were determined. FTIR and SEM were also applied to examine cell wall modifications of AMHRCs following IPC treatment. To the best of our knowledge, there is no report on the utilization of immobilized fungi both as biocatalyst and as elicitor to enhance secondary metabolite production in live plant *in vitro* cultures.

## Results and discussion

### Feasibility of IPC treatment for promoting AG IV production in AMHRCs

Biotransformation technology that possesses chemo‐, regio‐, and stereoselective reaction characteristics is deemed economically and ecologically competitive in the synthesis of desired compounds/products that are difficult or impossible to be obtained through conventional chemical procedures (Groussin and Antoniotti, 2012; Hegazy *et al*., 2015). The recognition of microorganisms as promising biocatalysts has taken on greater significance in the production of chemicals, pharmaceuticals and food ingredients (Borges *et al*., 2009; Cao *et al*., 2015; Hegazy *et al*., 2015). Fungi are always considered as an inexhaustible source of novel biocatalysts with numerous applications, especially for fungal endophytes (Borges *et al*., 2009; Zeng *et al*., 2014). In recent years, there is an increasing appreciation of the vast repertoire of extracellular enzymes (e.g. laccase, cellulases, lipases, proteases, amylases, chitinases, tannase, glucosidases, asparaginase and pectinase) secreted from fungal endophytes, which motivates bioprospecting these organisms to screen novel and efficient biocatalysts that might help address challenges in medicine, food security, energy production and environmental quality (Suryanarayanan *et al*., 2012). It is therefore not surprising that *P. canescens* is found to possess an excellent deacetylation function that can effectively transforms other AG derivatives to AG IV (Yao *et al*., 2014).

Currently, immobilization of microorganisms by Ca‐alginate gel (CAG) has emerged as an ideal method that is frequently used in biotransformation procedures for the production of biologically active compounds (Chen *et al*., 2013; Feng *et al*., 2015; Jin *et al*., 2013; Yao *et al*., 2014). This provides such bioprocess scale‐up feasibility and biocatalyst reusability with high operational stability. Previously, the fermentation of *A. membranaceus* dried roots with IPC can achieve the biotransformation of some AG IV precursors to AG IV with high‐efficiency (Yao *et al*., 2014). However, whether IPC can transform these precursors to AG IV in live plant *in vitro* cultures, *that is* AMHRCs, should be evaluated in this work.

As previously described, 36‐day‐old AMHRCs (mid‐stationary growth stage) originated from a high‐productive hairy root line (AMHRL VI) are capable of producing the maximum levels of different AG derivatives (including AG I, AG II, IAG II and AG IV) (Jiao *et al*., 2015). For promoting AG IV production to the maximum extent under IPC treatment, 36‐day‐old AMHRCs were therefore adopted as the optimal culture system in this work. To evaluate the feasibility of IPC treatment for promoting AG IV production, 36‐day‐old AMHRCs were initially challenged with IPC through 2 days of co‐cultivation. As shown in Figure 2e, IPC‐treated AMHRCs produced much more AG IV (1.585 ± 0.106 mg/g DW) in comparison with nontreated AMHRCs (0.187 ± 0.014 mg/g) and CAG‐treated AMHRCs (0.196 ± 0.009 mg/g). On the contrary, yields of AG IV precursors (0.488 ± 0.033 mg/g DW of AG I, 0.445 ± 0.016 mg/g DW of AG II and 0.111 ± 0.009 mg/g DW of IAG II) in IPC‐treated AMHRCs were much lower than those in nontreated AMHRCs (1.352 ± 0.091 mg/g DW of AG I, 0.735 ± 0.042 mg/g DW of AG II, and 0.191 ± 0.007 mg/g DW of IAG II) and CAG‐treated AMHRCs (1.366 ± 0.048 mg/g DW of AG I, 0.741 ± 0.017 mg/g DW of AG I,I and 0.182 ± 0.019 mg/g DW of IAG II). Additionally, no significant differences were observed in the yields of all AG derivatives between nontreated AMHRCs and CAG‐treated AMHRCs, suggesting that the immobilization supporter (CAG) could not affect AG production in AMHRCs. As expected, the applied IPC treatment was indeed feasible for the deacetylation of these precursors (AG I, AG II and IAG II) to form AG IV in AMHRCs, which would realize the enhanced and sustainable production of AG IV in plant *in vitro* cultures.

### Time course of IPC deacetylation biocatalysis in AMHRCs

**Figure 3:**
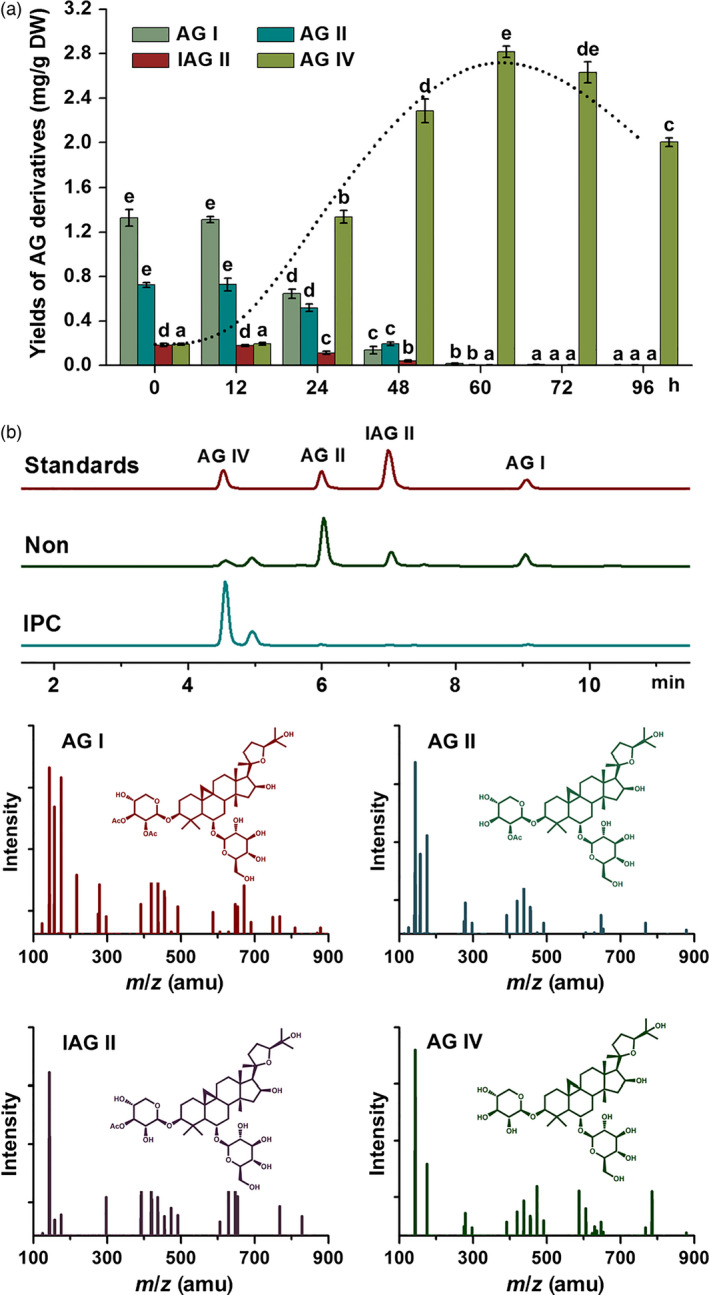
(a) Biotransformation time course of different AG derivatives (the dashed line marking the kinetic curve of AG IV production) in 36‐day‐old AMHRCs (*ca*. 10^3^ spores/mL, co‐cultivation temperature 30 °C and initial pH value of media 7.0); (b) representative LC‐MS/MS with SRM total ion chromatograms of standards and extracts from nontreated and IPC‐treated AMHRCs. Non, nontreated AMHRCs; IPC, IPC‐treated AMHRCs. Mean ± SD values not sharing the same lowercase letters are significantly different (*P* < 0.05).

To obtain the appropriate time for IPC deacetylation biocatalysis in AMHRCs under the predetermined parameters (*ca*. 10^3^ spores/mL, co‐cultivation temperature 30 °C and initial pH value of media 7.0 shown in Figure S1), yields of AG IV and its precursors (AG I, AG II and IAG II) in samples harvested at different co‐cultivation time points (0, 12, 24, 48, 60, 72 and 96 h) were monitored. As exhibited in Figure 3a, all three precursors were found to be almost transformed to AG IV after 60 h. During this process, an interesting phenomenon was observed that AG I was transformed faster than AG II and IAG II. More specifically, the decline rate of AG I yield (50.88%) from 12 to 24 h was much higher as against those of AG II (29.16%) and IAG II (37.08%). Factually, AG II and IAG II have one extra acetyl residue attached at C‐3 position but AG I possesses tow (Figure 1). Theoretically, the deacetylation of AG II and IAG II was simpler relative to AG I, as they could be directly converted to AG IV *via* one‐step hydrolysis. It is inferred that AG II and IAG II were likely to be two intermediates during the deacetylation process of AG I. Similar results were reported by Zhou *et al*. (2014), who found that the transformation of AG I to AG IV by a novel acetyl esterase was a two‐stage hydrolysis step, in which formation of AG II (majority) and IAG II (minority) in the first phase followed by conversion of the two intermediates to AG IV in the second stage. Also, it is confirmed that *P. canescens* possesses the similar acetyl esterase that can perform the deacetylation function. Studies are underway to purify this enzyme from *P. canescens* to facilitate the biotransformation process in the future.

Additionally, the dashed line in Figure 3a demonstrated that AG IV yield significantly increased during the initial period of 12–48 h, reached the peak value at 60 h and gradually decreased afterwards. Factually, microorganisms are capable of continuously producing a great variety of enzymes in a short period of time due to their natural characteristic to multiply (Hegazy *et al*., 2015). The decreased yield of AG IV after 60 h could be ascribed to the emergence of some adverse enzymes that can transform AG IV to other metabolites. Accordingly, it is deduced that the appropriate co‐cultivation time (60 h) could realize the balance between the desired deacetylation and the adverse bioconversion for the maximal production of AG IV in IPC‐treated AMHRCs. Under the optimal time point, IPC‐treated AMHRCs was capable of producing AG IV up to 2.816 ± 0.052 mg/g DW, which tremendously increased 14.59‐fold as against that of nontreated control (0.193 ± 0.007 mg/g DW). Also, LC‐MS/MS chromatograms confirmed the IPC biotransformation of these precursors (AG I, AG II and IAG II) to AG IV in AMHRCs after 60 h (Figure 3b).

**Figure 4:**
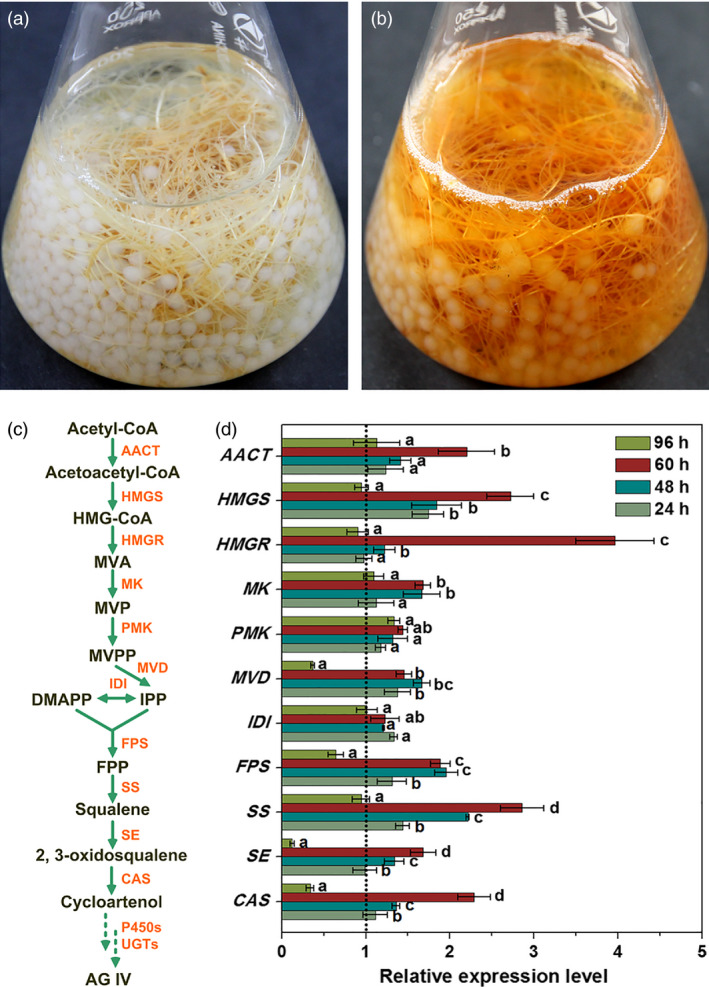
Phenotypes of AMHRCs before (a) and after (b) IPC treatment; (c) AG IV biosynthetic pathway in *A. membranaceus*; (d) transcriptional levels of AG IV biosynthetic genes in IPC‐treated AMHRCs at different time points (24, 48, 60 and 96 h). AACT, acetoacetyl‐coenzyme A (CoA) thiolase; CAS, cycloartenol synthase; CYP450s, cytochrome P450‐dependent monooxygenases; DMAPP, dimethylallyl diphosphate; FPP, farnesyl pyrophosphate; FPS, farnesyl diphosphate synthase; HMGR, 3‐hydroxy‐3‐methylglutaryl coenzyme A (HMG‐CoA) reductase; HMGS, HMG‐CoA synthase; IDI, iso‐pentenyl diphosphate isomerase; IPP, isopentenyl diphosphate; MVA, mevalonate; MVD, mevalonate diphosphate decarboxylase; MVPP, 5′‐diphosphomevalonate; MVP, 5′‐phosphomevalonate; MK, mevalonate kinase; PMK, phosphomevalonate kinase; SE, squalene epoxidase; SS, squalene synthase; UGTs, uridine diphosphate‐dependent glycosyltransferases. Mean ± SD values not sharing the same lowercase letters are significantly different (*P* < 0.05).

Theoretically, the resulting AG IV yield should be 2.291 mg/g DW that was calculated from the bioconversion of three precursors (AG I, AG II and IAG II). Most interestingly, the actual AG IV yield (2.816 mg/g DW) was significantly higher than the forecasted value (2.291 mg/g DW) in this work. On one hand, this may be due to the transformation of other minor AG derivatives with acetyl residues (e.g. acetylastragaloside I, isoastragaloside I, agroastragaloside III) to AG IV by IPC deacetylation. On the other hand, IPC as a biotic elicitor can trigger plant defence metabolisms, thus resulting in the further enhanced accumulation of AG IV (function as phytoalexin) in AMHRCs. Moreover, it is worth mentioning that AMHRCs exhibited a dark yellow colour (browning colour) in hairy root tissues after 60 h of IPC treatment (Figure 4b) in contrast to the white root tissues before treatment (Figure 4a). This phenomenon was a significant indication of stress/elicitation response in AMHRCs after IPC treatment. In regard to this, it is interesting to further study for deciphering the exact molecular events undergoing IPC elicitation of AG IV biosynthesis.

### Biosynthetic gene expressions underlying IPC elicitation in AMHRCs

Due to the pathogenic nature of microorganisms, the constituents and secretions of fungi are always able to induce hypersensitive responses in plant cells, which can activate plant secondary metabolism involved in defence and thereby enhance phytoalexin biosynthesis (Baldi *et al*., 2009). To clarify the molecular mechanism underlying IPC elicitation, the expression of eleven genes encoding enzymes involved in AG IV biosynthetic pathway (Figure 4c) was determined by qRT‐PCR. In this work, hairy root samples obtained from IPC‐treated AMHRCs were taken for qRT‐PCR analysis at 24, 48, 60 and 96 h post‐treatment. The relative expression levels of all investigated genes were normalized using the internal reference *18S* gene and as compared to control.

As displayed in Figure 4d, all tested genes were observed to up‐regulate during the period of IPC treatment from 24 to 60 h, suggesting that IPC could indeed activate the transcription of these biosynthetic genes, thus promoting AG IV accumulation in AMHRCs. Among them, expression levels of *AACT*,* HMGS*,* HMGR*,* MK*,* PMK*,* SS*,* SE* and *CAS* genes were found to be gradually stimulated, and reached their peak values at 60 h necessary for the maximal AG IV production. Particularly, the highest transcriptional level of *HMGR* gene at 60 h was 5.46‐fold higher relative to control. Generally, HMGR is well recognized to be the most important rate‐limiting enzyme in the triterpene biosynthetic pathway (Kim *et al*., 2013, 2014; Laule *et al*., 2003). In this study, the significant induction of *HMGR* transcription by IPC treatment was undoubtedly beneficial for the enhanced AG IV biosynthesis in AMHRCs. Moreover, it should be pointed out that the expression levels of some biosynthetic genes (*MVD*,* FPS*,* SE* and *CAS*) were considerably repressed at 96 h compared to control. The prolonged co‐cultivation time might lead to the excessive elicitation that would cause the metabolic damage or in extreme cases the death of hairy roots. This also can partly explain the decline trend of AG IV yield along a time course from 60 to 96 h (Figure 3a).

### Cell wall modifications of AMHRCs following IPC treatment

As FTIR spectroscopy can provide the useful information for identifying the presence of functional groups or chemical bonds in an interaction system, it was applied in this study for studying the modifications in cell walls of *A. membranaceus* hairy roots between control and IPC‐treated sample. In comparison with the control spectrum, the characteristic absorbance bands of lignin at 1254 cm^−1^ (guaiacyl/syringyl ring and C–O stretching vibration), 1512 cm^−1^ (aromatic skeletal vibration) and 1602 cm^−1^ (symmetric aromatic ring C=C stretching vibration) were more intense in IPC‐treated sample, which indicated that lignin was enriched in hairy root tissues after IPC treatment (Figure S2A). Additionally, the intensity of the characteristic peak of cellulose and hemicellulose at 1385 cm^−1^ (C–H bending vibration) decreased in the spectrum of IPC‐treated sample, which was also ascribed to the relatively increased content of lignin (Figure S2A). Generally, plants suffering from various biotic and abiotic stresses (wounding, pathogen infection, UV radiation, hyperosmotis, etc.) can induce the excessive biosynthesis of lignin in a short time and promote cell wall lignification at the injury site, thus resulting in the improved defence ability against the damages caused by external stresses (Tronchet *et al*., 2010; Vanholme *et al*., 2010). In this work, the enhanced accumulation of lignin provided the evidence that *A. membranaceus* hairy roots were indeed elicited by IPC treatment.

Meanwhile, triterpene saponins (e.g. AGs) act as defence secondary metabolites that can also be inducibly synthesized, transferred and enriched in cell walls to improve the survival of plant cells/organs under pathogenic or environmental stresses (Augustin *et al*., 2011; Khakimov *et al*., 2015; Moses *et al*., 2015). As inferred, the acetyl esterase secreted from IPC would be driven to the surface of hairy root tissues due to the chemotaxis characteristic (i.e. the enrichment of AG substrates in cell walls). Factually, the relative enhancement in characteristic adsorption bands of protein at 1530 cm^−1^ (the C=O bonds in amide I) and 1635 cm^−1^ (the combination of C–N stretching and N–H vibration in amide II backbone) in IPC‐treated sample could support this inference (Figure S2A). Moreover, SEM examination was performed to give further insight on the surface morphology of samples. The micrograph of control (Figure S2B) represented a compact structure with abundant root hairs (arrows). However, it was clearly observed that the surface of IPC‐treated sample (Figure S2C) showed highly roughness in all directions with some attached substances (arrows), which also provided a supporting evidence of the biotransformation phenomena.

### Stability and reusability of IPC beads in AMHRCs

**Figure 5:**
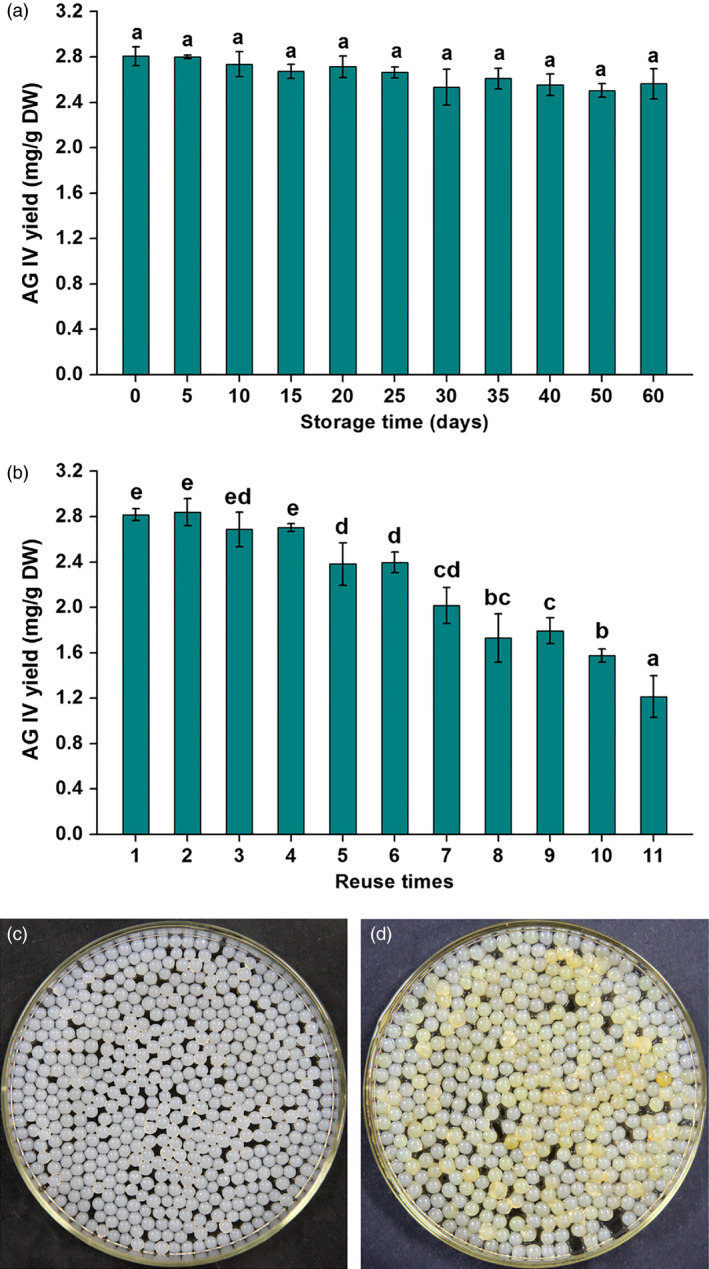
(a) Effect of IPC beads stored at 4 °C on AG IV production during the period of 60 days; (b) effect of the recovered IPC beads on AG IV production in repeat‐batch cultures; photographs of IPC beads before (c) and after six cycles (d). Mean ± SD values not sharing the same lowercase letters are significantly different (*P* < 0.05).

Generally, the stability and reusability of immobilized microorganisms are crucially important for their industrial applications (Cappa *et al*., 2014). In this study, the preprepared IPC beads were stored at 4 °C under sterile conditions, and taken out to co‐culture with AMHRCs at regular intervals during the storage period of 60 days. The stability of IPC was evaluated by monitoring AG IV yield in AMHRCs at 60 h post‐treatment. As shown in Figure 5a, the AG IV yield had no significant change during the IPC storage period and remained around the optimal value (2.816 mg/g DW), indicating that IPC possessed the excellent stability and could keep its inherent deacetylation activity and elicitation effect during the long storage time. Additionally, IPC beads could be easily separated from AMHRCs and reused in this work, which would greatly decrease the operational cost for practical application. Accordingly, the reusability of IPC was tested by determining AG IV yield in AMHRCs after 11 successive batches. As shown in Figure 5b, the recovered IPC beads could be reused six cycles, and about 85% of the initial AG IV yield (2.395 ± 0.091 mg/g DW) was still obtained in the sixth run. Moreover, photographs of IPC beads before (Figure 5c) and after six cycles (Figure 5d) exhibited that the recovered IPC beads could still maintain the integrity of ball shape but except for the slightly bigger volume and a little deeper colour. Although IPC possessed the acceptable reusability performance, further improvements in the immobilization step using different polymers (e.g. chitosan and polyethylenimine) would be done to add to the possible economic values of the overall process.

## Conclusions

AG IV is a scarcer bioactive compound with multiple pharmacological activities. The novelty of this work was to establish a co‐cultivation system of immobilized fungus (IPC) and plant *in vitro* cultures (AMHRCs), in which IPC could possibly perform both deacetylation biocatalysis and elicitation effect for promoting AG IV production. AMHRCs (36 days old) co‐cultured with IPC for 60 h resulted in the maximum AG IV enhancement (up to 14.59‐fold). Moreover, IPC beads possessed excellent stability and satisfactory reusability for industrial applications. Expectantly, the commercial scale‐up of AG IV production at bioreactor level using the proposed biotechnology approach seems feasible and promising.

## Experimental procedures

### Plant materials and reagents

The immobilization of *P. canescens* spores (Figure 2b) in CAG beads was prepared according to the method described by Yao *et al*. (2014). AMHRCs (Figure 2d) were initiated by culturing a high‐productive hairy root line (AMHRL VI) (Figure 2c) under the optimal conditions as reported by Jiao *et al*. (2015). AG standards including AG I, AG II, IAG II and AG IV were purchased from Weikeqi Biological Technology Co. Ltd. (Sichuan province, China). MiniBEST Plant RNA Extraction Kit, PrimeScript^™^ RT reagent Kit and SYBR Premix Ex Taq^™^ II Kit were provided by TaKaRa (Dalian, China). Other reagents of either analytical or optical grades were purchased from Beijing Chemical Reagents Co. (Beijing, China). Ultrapure water was prepared from a Milli‐Q system at 18.3 M resistance (Millipore, Bedford, MA).

### Co‐cultivation of AMHRCs and IPC

During experiments, IPC beads were transferred into a series of flasks containing 36‐day‐old AMHRCs with 100 mL of fresh MS media. And, the biomass concentration of 36 day‐old hairy roots was 15.45 ± 0.81 g/L, DW basis. Based on results of preliminary experiments (Figure S1), the spore amount of IPC load (*ca*. 10^3^ spores/mL), co‐cultivation temperature (30 °C) and initial pH value of media (7.0) were selected as the optimal operational parameters regarding AG IV production. Subsequently, these cultures were incubated under darkness on an orbital shaker at 100 rpm and 30 °C, and harvested under a series of time points (0, 12, 24, 48, 60, 72 and 96 h). Meanwhile, nontreated AMHRCs and CAG‐treated AMHRCs (addition of CAG beads without fungus spores) were the two groups of control cultures. After co‐cultivation, IPC beads were simply recovered by filtration, rinsed with sterile water and utilized for the next time to evaluate their reusability. Root samples were collected, washed by distilled water and divided into two parts: one being frozen immediately with liquid nitrogen and stored at −80 °C for the RNA isolation, and the others being dried in a vacuum oven at 60 °C until the constant dry weight (DW) for the liquid–solid extraction of AGs. Also, medium samples were collected for the liquid–liquid extraction of AGs.

### AG extraction and LC‐MS/MS analysis

Extraction of AGs from root and media samples was performed as previously described (Jiao *et al*., 2016). All extracts were evaporated to dryness at 45 °C using a rotary evaporator, re‐dissolved in methanol and filtered through a 0.45‐μm membrane prior to LC‐MS/MS analysis. According to our previous report (Jiao *et al*., 2015), an established LC‐MS/MS method with selected reaction monitoring (SRM) was used to simultaneously determine the target AG derivatives in AMHRCs. Different ion combinations of SRM at *m/z* 869.9 → 143.1, *m/z* 827.4 → 143.1 and *m/z* 785.8 → 143.1 with highest signal intensities were selected for the identification and quantification of AG I, AG II & IAG II, and AG IV, respectively. The content of individual target compound was calculated by the corresponding calibration curve and reported as microgram per gram based on the DW of roots.

### qRT‐PCR analysis

Total RNA extraction and cDNA preparation were performed following the corresponding kits' instructions. qRT‐PCR analysis was carried out using a Stratagene Mx3000P Real‐Time PCR system (Agilent Technologies, Santa Clara, CA). Specific primers of associated genes (Table S1) were designed as suggested previously (Jiao *et al*., 2016). The reaction solution for qRT‐PCR assay was prepared in accordance with the kit's protocols. The amplification procedure comprised an initial denaturation at 95 °C for 3 min, followed by 40 cycles of denaturation at 95 °C for 15 s, annealing at 55 °C for 30 s and extension at 72 °C for 20 s. *18S* gene was utilized as the internal reference, and the relative expression level was analysed using the delta CT method (Livak and Schmittgen, 2001).

### FTIR spectroscopy

FTIR spectroscopy was recorded in the frequency range of 4000–400 cm^−1^ using an Affinity‐1 spectrophotometer (Shimadzu, Japan). The dry root samples (1%) were ground with KBr powder (spectroscopic grade) and pressed into discs prior to spectra measurement. The data were collected, corrected for the background and plotted as transmittance (%) in function of the wavelength (cm^−1^).

### SEM observation

SEM observation was conducted on a Quanta‐200 environmental scanning electron microscope system (FEI Company, Hillsboro, OR, USA). The dry root samples were fixed on a specimen holder with an aluminium tape and sputtered with a thin layer of gold prior to micrograph examination. The SEM was run under high vacuum condition at an accelerating voltage of 12.5 kV.

### Statistical analysis

All experiments were performed in triplicates, and results were expressed as means ± standard deviations. Differences between multiple groups of data were determined by one‐way ANOVA with Tukey's test on the significant level declared at *P* < 0.05. All statistical analyses were performed using the SPSS statistical software 17.0 (SPSS Inc, Chicago, IL).

## Supporting information


**Figure S1** (A) Effect of spore amount of IPC load on AG IV production (co‐cultivation temperature 28 °C and initial pH value of media 5.8); (B) Effect of co‐cultivation temperature on AG IV production (*ca*. 10^3^ spores/mL, initial pH value of media 5.8 and time 48 h); (C) Effect of initial pH value of media on AG IV production (*ca*. 10^3^ spores/mL, co‐cultivation temperature 30 °C and time 48 h). Mean ± SD values not sharing the same lowercase letters are significantly different (*P *< 0.05).
**Figure S2** (A) FTIR spectrum analysis between control and IPC‐treated sample; SEM micrographs of control (B) and IPC‐treated sample (C). Control, nontreated AMHRCs.
**Table S1** Primers of genes involved in AG IV biosynthetic pathway.
