# Supplementary material for: Deacetylation biocatalysis and elicitation by immobilized Penicillium canescens in Astragalus membranaceus hairy root cultures: towards the enhanced and sustainable production of astragaloside IV
Source: Plant Biotechnol J. 2016 Sep 16;15(3):297–305. doi: 10.1111/pbi.12612 (PMC5316919; doi:10.1111/pbi.12612)
Supplement: Supplementary file 1 — Figure S1 (A) Effect of spore amount of IPC load on AG IV production (co‐cultivation temperature 28 °C and initial pH value of media 5.8); (B) Effect of co‐cultivation temperature on AG IV production (ca. 103 spores/mL, initial pH value of media 5.8 and time 48 h); (C) Effect of initial pH value of media on AG IV production (ca. 103 spores/mL, co‐cultivation temperature 30 °C and time 48 h). Mean ± SD values not sharing the same lowercase letters are significantly different (P < 0.05). Figure S2 (A) FTIR spectrum analysis between control and IPC‐treated sample; SEM micrographs of control (B) and IPC‐treated sample (C). Control, nontreated AMHRCs. Table S1 Primers of genes involved in AG IV biosynthetic pathway. [file PBI-15-297-s001.doc]

**Supplementary data to:**

**Deacetylation biocatalysis and** **elicitation** **by immobilized *Penicillium canescens* in *Astragalus membranaceus* hairy root cultures: towards the enhanced and sustainable production of astragaloside IV**

Qing-Yan Gai ^1,2,3,#^, Jiao Jiao ^1,2,3,#^, Meng Luo ^1,2,3^, Wei Wang ^1,2,3^, Li-Ping Yao ^1,2,3^, Yu-Jie Fu ^1,2,3^,*

^1^ *Key Laboratory of Forest Plant Ecology, Ministry of Education, Northeast Forestry University,* *Harbin 150040, PR China*

^2^ *Engineering Research Center of Forest Bio-Preparation, Ministry of Education, Northeast Forestry University, Harbin 150040, PR China*

^3^ *Collaborative Innovation Center for Development and Utilization of Forest Resources, Harbin, Heilongjiang 150040, PR China*

* Corresponding authors: Y.-J. Fu

Tel./Fax: +86-451-82190535; E-mail: yujie_fu2011@yahoo.com, yujie_fu@163.com.

^#^ These authors contributed equally to this work.


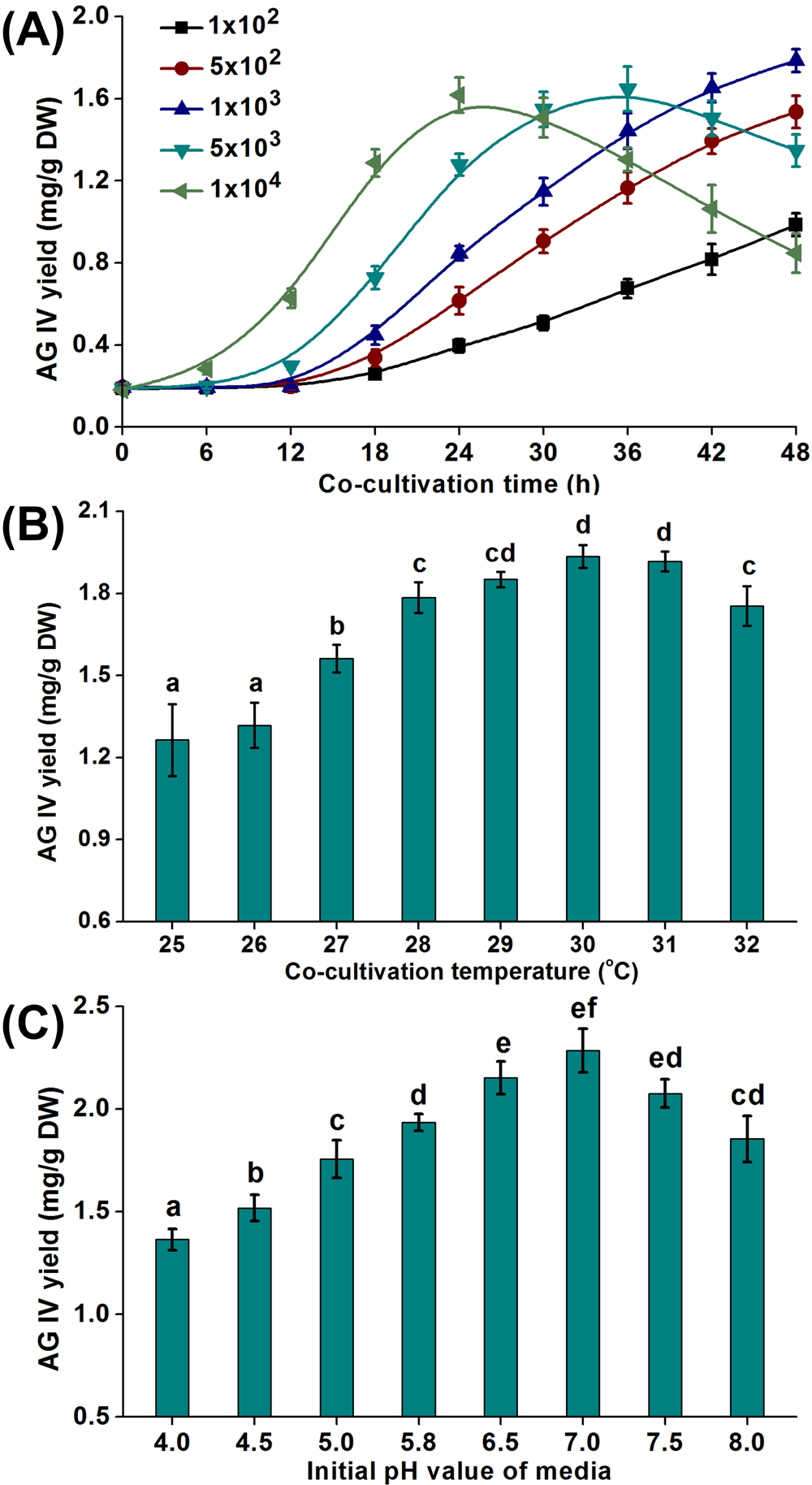


**Fig. S1.** (A) Effect of spore amount of IPC load on AG IV production (co-cultivation temperature 28 °C and initial pH value of media 5.8); (B) Effect of co-cultivation temperature on AG IV production (*ca.*10^3^ spores/mL, initial pH value of media 5.8 and time 48 h); (C) Effect of initial pH value of media on AG IV production (*ca.*10^3^ spores/mL, co-cultivation temperature 30 °C and time 48 h). Mean ± SD values not sharing the same lowercase letters are significantly different (*P* < 0.05).


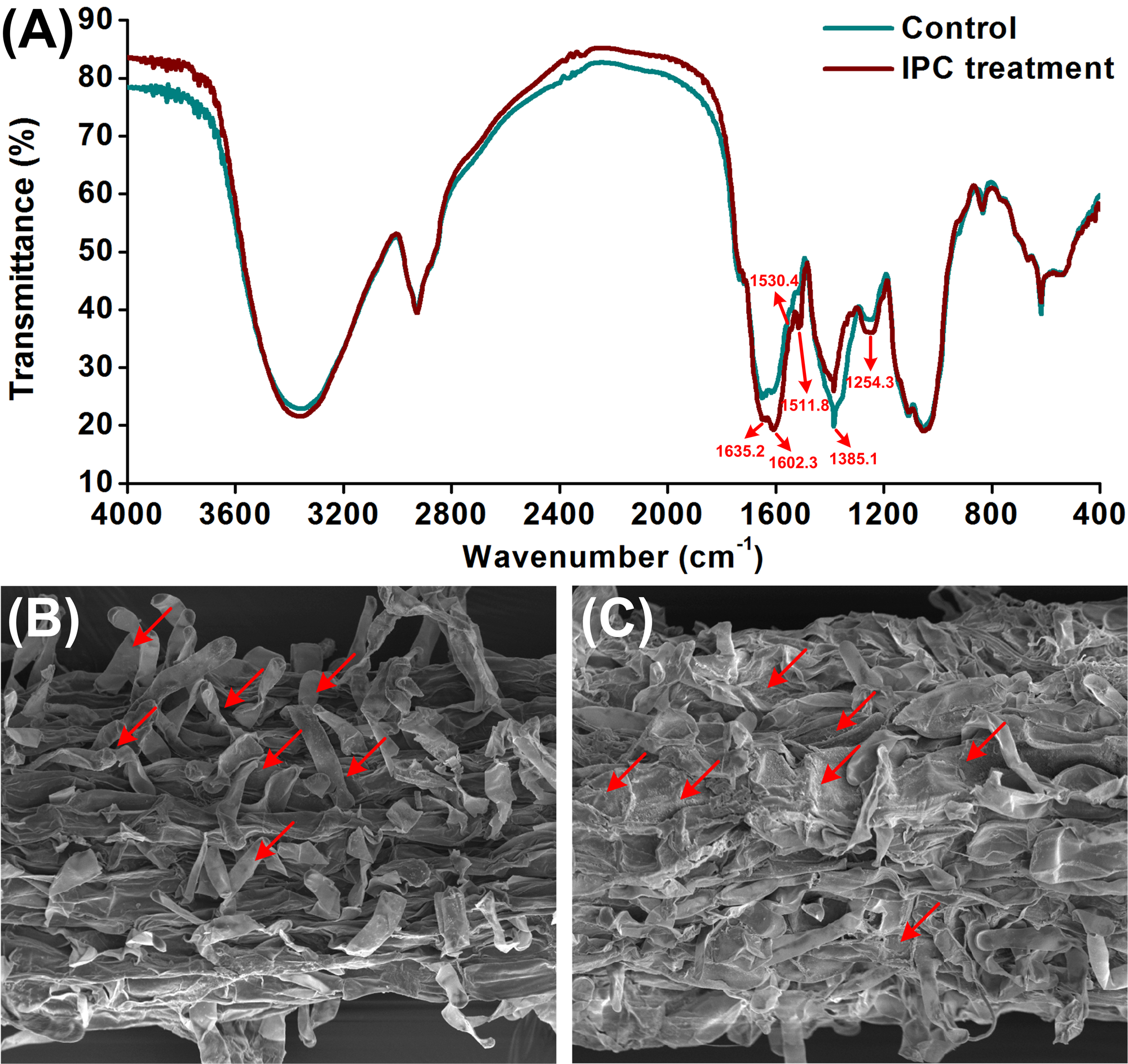


**Fig. S2.** (A) FTIR spectrum analysis between control and IPC-treated sample; SEM micrographs of control (B) and IPC-treated sample (C). Control, non-treated AMHRCs.

**Table S1.** Primers of genes involved in AG IV biosynthetic pathway.

| Gene names | Primer sequences (5′ to 3′) | Product sizes (bp) |
| --- | --- | --- |
| *AACT* | Forward: GGTGAGCGGAGAGAAGGCAT | 110 |
|  | Reverse: CGAGTGCTGGAGCGGTTGTA |  |
| *HMGS* | Forward: CCTTCTTCGGCATTGCTTTCATC | 127 |
|  | Reverse: TCGAGATCCCGGCTTTGGTA |  |
| *HMGR* | Forward: GCCGGCCACCATAAACGA | 155 |
|  | Reverse: CGACGGAGAAGAAGAGGGTGAA |  |
| *MK* | Forward: AACATGCCGTTGTTCACGGA | 139 |
|  | Reverse: AACTCCAATGCCGCATCGTT |  |
| *PMK* | Forward: AGATCACCCGGACAGGAAGGA | 142 |
|  | Reverse: CCGCACATAGCGATGACTTCC |  |
| *MVD* | Forward: TAAGGGAGATCCGCGCTCGT | 144 |
|  | Reverse: CAGCTGACGAAGCCAGTCCA |  |
| *IDI* | Forward: TGCTGGTGAGGGAGGTTTGAA | 116 |
|  | Reverse: TCATGTCAGCGACCTCACCAA |  |
| *FPS* | Forward: CGACCGGATGCTGGACTACA | 136 |
|  | Reverse: CCAACCAAGAGCACTGGCAA |  |
| *SS* | Forward: AAGCAGATCCCTCCGGAACC | 113 |
|  | Reverse: ACAGCGTTGCGAAGTTCGGT |  |
| *SE* | Forward: TGGAACAAGGAACCGTGACATCT | 150 |
|  | Reverse: ACAAAGAGAACGCCTCAAGTTGGA |  |
| *CAS* | Forward: TGGAGATTTCCCACAGCAGGA | 150 |
|  | Reverse: CAAGTTGCGGCATTTGGTGT |  |
| *18S* | Forward: TGCAGAATCCCGTGAACCATC | 104 |
|  | Reverse: AGGCATCGGGCAACGATATG |  |
